# Supplementary material for: Complex‐centric proteome profiling by SEC‐SWATH‐MS
Source: Mol Syst Biol. 2019 Jan 14;15(1):e8438. doi: 10.15252/msb.20188438 (PMC6346213; doi:10.15252/msb.20188438)
Supplement: Supplementary file 8 — Dataset EV7 [file MSB-15-e8438-s008.zip › feature_plots_string/P46087.pdf]

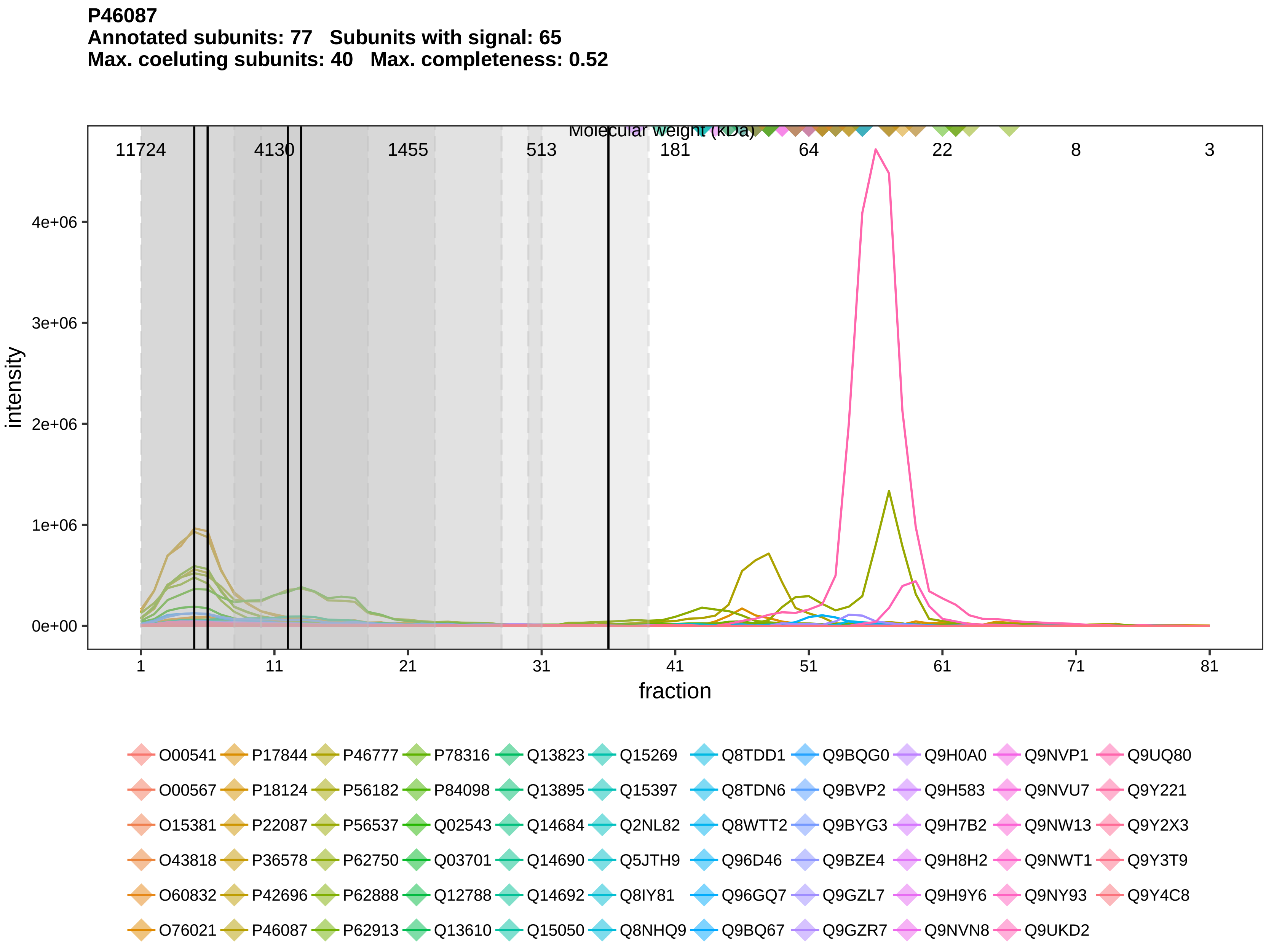
P46087
Annotated subunits: 77 Subunits with signal: 65
Max. coeluting subunits: 40 Max. completeness: 0.52
Molecular weight (kDa)
11724
4130
1455
513
181
64
22
8
3
intensity
4e+06
3e+06
2e+06
1e+06
0e+00
fraction
1
11
21
31
41
51
61
71
81
O00541
P17844
P46777
P78316
Q13823
Q15269
Q8TDD1
Q9BQG0
Q9H0A0
Q9NVP1
Q9UQ80
O00567
P18124
P56182
P84098
Q13895
Q15397
Q8TDN6
Q9BVP2
Q9H583
Q9NVU7
Q9Y221
O15381
P22087
P56537
Q02543
Q14684
Q2NL82
Q8WTT2
Q9BYG3
Q9H7B2
Q9NW13
Q9Y2X3
O43818
P36578
P62750
Q03701
Q14690
Q5JTH9
Q96D46
Q9BZE4
Q9H8H2
Q9NWT1
Q9Y3T9
O60832
P42696
P62888
Q12788
Q14692
Q8IY81
Q96GQ7
Q9GZL7
Q9H9Y6
Q9NY93
Q9Y4C8
O76021
P46087
P62913
Q13610
Q15050
Q8NHQ9
Q9BQ67
Q9GZR7
Q9NVN8
Q9UKD2
